# Factors and Radiographic Findings Influencing Patient-Reported Outcomes Following Maisonneuve Fractures

**DOI:** 10.7759/cureus.43536

**Published:** 2023-08-15

**Authors:** Thomas Sanchez, Turner Sankey, Mila B Scheinberg, Samuel Schick, Swapnil Singh, Naga Cheppalli, Chandler Davis, Ashish Shah

**Affiliations:** 1 Orthopaedic Surgery, University of Alabama at Birmingham, Birmingham, USA; 2 Orthopaedics, VA Hospital Albuquerque, Albuquerque, USA

**Keywords:** post-operative review ankle fracture, ankle radiographic study, lateral malleolus fracture, medial malleolus fracture, patient-reported outcomes, promis scores, syndesmosis, ankle fracture, maisonneuve fracture

## Abstract

Background: This research adds to the literature by providing prognostic information for physicians and patients regarding the outcomes of operative management of Maisonneuve fractures (MFs). To date, this is the only cohort study of patient-reported outcomes measurement information systems (PROMIS) scores following surgical fixation of MF. Patient outcomes were compared focusing on the mean population with an inter-analysis using basic demographic information, radiographic findings, and patient comorbidities and their respective impact on PROMIS scores.

Methods: A total of 24 patients between 2012 and 2020 met the inclusion criteria and completed PROMIS surveys at a minimum of 18 months postoperatively. Patient charts were reviewed through the electronic medical record (EMR) for demographic information and comorbidities as well as operative variables. PROMIS scores for physical function (PF), pain interference (PI), and depression were obtained via follow-up visits and phone calls. The impact of categorical variables on complications was compared using Chi-Squared tests. Variables were analyzed with a type 3 SS test to stratify independent risk factors’ effect on PROMIS scores and to account for confounding variables.

Results: PROMIS PF averaged 44.84 and was significantly affected by BMI>30 (p=.033), hypertension (HTN) (p=.026), patients with clinical anxiety or depression (p=.047), and subsequent screw removal (p=.041). PROMIS PI averaged a score of 54.57 and was significantly affected by BMI>30 (p=.0046), coronary artery disease (CAD) (p=.0123), patients with clinical anxiety or depression (p=.0206), and subsequent screw removal (p=.0039). PROMIS depression scores averaged 46.03 and were significantly affected by the presence of CAD (p=.049) and subsequent screw removal (p=.023).

Conclusion: Patient-reported outcomes following MF surgery demonstrated PROMIS scores within +/- 1 standard deviation of the population-based control, and thus many patients can reasonably expect to return to a level of function comparable to the general population. Nonetheless, the significant effects of patient comorbidities and surgical variables ought to be evaluated and utilized as prognostic indicators when managing patient expectations prior to operative treatment of an MF injury.

## Introduction

Ankle fractures are common injuries managed by orthopedic surgeons with an incidence of approximately 180 fractures per 100,000 people per year [1]. Ankle injuries with an associated Maisonneuve fracture (MF) component are not as frequent and comprise around one to seven percent of all ankle fractures [2]. MFs comprise a wide variety of injuries including Maisonneuve variants with proximal tibiofibular (TF) dislocation or two-level fibular fractures. It is important to identify the distinction between a classical Weber type-C fracture vs an MF, which is a special subtype of a Weber C fracture. The main component that differentiated the two is that that the fracture of the fibula has to be demonstrated within the proximal third of the fibula shaft in an MF. MFs are a result of torsional injury to the foot and ankle with characteristic high fibular fracture, disruption of the syndesmotic ligaments, interosseous membrane, and often have associated medial and posterior malleolar fractures. These injuries are often missed, and appropriate diagnosis requires extensive history, physical exam, and imaging. Decreased push-off power, tenderness over anterior inferior tibiofibular ligament (AITFL), gross soft tissue damage, and tenderness over the proximal fibula are all findings that indicate a syndesmotic injury and potential MF [3]. The external rotation squeeze test is a crucial test that is very specific for identifying the pathology of the syndesmosis [4]. Weight-bearing radiographs of the ankle should be obtained, if possible, with additional knee films if an MF is suspected. Advanced imaging can be beneficial to rule out any concomitant pathologies.

MFs are classified based on the Lauge-Hansen system as pronation-external rotation injuries or Danis-Weber class C injuries [2]. Patients can expect to obtain satisfactory outcomes following appropriate management of both the bony and syndesmotic injuries. However, further studies are needed to demonstrate the true efficacy of the varying treatment modalities.

Recently, computer adaptive tests (CAT) have become the standard for patient-reported outcomes due to their adaptability and individualization. The National Institutes of Health (NIH)-supported patient-reported outcomes measurement information system (PROMIS) has been validated in a variety of orthopedic conditions as a superior method to assess pain, physical function (PF), mental health status, symptoms, and overall outcomes [5]. Support for PROMIS is growing due to decreased time to complete, increased generalizability, and removal of the floor/ceiling effect [2]. Gausden et al. concluded that PROMIS testing was superior to legacy foot and ankle outcome scores for patients after ankle fracture surgery in its ability to stratify significant changes at different time points in patients’ recovery [5].

The study’s primary aim is to evaluate factors influencing patient-reported PROMIS pain interference (PI), PF, and depression scores following operative fixation of MF. In addition, we describe the influence of concomitant injuries, fixation method, and quality of reduction on the aforementioned PROMIS scores.

## Materials and methods

After institutional review board approval, all patients undergoing syndesmotic fixation (CPT 27829) at a tertiary academic center between 2012 and 2020 were identified. 770 patients were initially identified, and radiographs were reviewed by a team of three foot and ankle fellowship-trained orthopedic surgeons to identify patients with AO-44C3 type MFs. Patients who did not complete the PROMIS survey, those with a minimum clinical follow-up of fewer than two years, radiological follow-up of fewer than six months, fracture patterns other than true MFs, and those under the age of 18 were excluded from the study. Radiographs were reviewed by a team of three foot and ankle fellowship-trained orthopedic surgeons. A total of 28 patients with AO-44C3 pattern fractures (3.6%) were identified.

Medical records were reviewed for patient characteristics and comorbidities. Concomitant injuries including medial malleolar fracture, posterior malleolar fracture, and deltoid ligament injury were recorded based on chart review. Operative variables assessed included the type of syndesmotic fixation (syndesmotic screw (SS) or suture button) and fixation of concomitant injuries. Postoperative complications record included persistent pain, wound complications, and the need for revision surgery. For patients who received SSs, the incidence of screw removal was assessed but was not considered a reoperation if planned.

Statistical analysis

An ANOVA was performed on the dataset to evaluate for preoperative patient variables with a significant effect on postoperative PROMIS scores. Assumptions were checked using the Shapiro-Wilk test for normality, describing a normally distributed cohort, and Q-Q plots. Tukey multiple comparisons were performed post hoc for significant F values (p<0.05) with three or more categories. Family-wise error rate was controlled with a Bernoulli correction. Post hoc power analysis found that in order to detect a PROMIS score difference of two, a minimum of 21 subjects was necessary for a power of 80% at an alpha of 0.05.

Patient-reported outcomes

The PROMIS PI version 1.1, PF version 1.2, and depression version 1.0 CATs are composed of 40-item, 12-item, and 29-item question banks, respectively. The algorithm of each CAT reduced the overall number of answered questions per domain by selecting the most relevant question based on the patient’s previous answer. The median population score for each category is 50 and higher scores correlate with increased association with each category. For example, a patient with a PI score of 60 has a pain score one standard deviation above the general population.

Patients were seen in follow-up visits or contacted over the phone and asked to complete PROMIS outcome surveys for PF, PI, and depression. 24 patients completed PROMIS outcomes measures, with a response rate of 86%. The 24 responses were obtained at a minimum of 24 months postoperatively.

## Results

A total of 24 patients met our inclusion criteria. Demographic and comorbidity frequency can be seen in Table 1.

**Table 1:** Patient Population Demographics and Treatment Type COPD: Chronic obstructive pulmonary disease; CAD: Coronary artery disease; HT: Hypothyroidism; HTN: Hypertension; SS: Syndesmotic screw

Demographics	Frequency n (%)
Male	16 (67%)
Female	8 (33%)
Age > 45	14 (58%)
BMI > 30	10 (42%)
ASA class 1 / 2	11 (46%)
ASA class 3 / 4	13 (54%)
Type 2 diabetes	5 (21%)
Peripheral neuropathy	0
Tobacco use	10 (42%)
HTN	10 (42%)
HT	1 (4%)
Rheumatoid arthritis	0
COPD	2 (8%)
CAD	3 (12.5%)
Osteoporosis/osteopenia	0
Psychiatric condition	8 (33%)
Posterior malleolus fx	8 (33%)
Medial malleolus fx	10 (42%)
Deltoid ligament injury	5 (21%)
Associated dislocation	4 (16%)
SSs	15 (62%)
Tightrope fixation	9 (38%)

The average PROMIS PF, PI, and depression scores are 44.84, 54.57, and 46.03, respectively (Table 2). PROMIS PF was independently affected by BMI >30 (f=7.57, p=.033), tobacco use (f=11.18, p=0.0029), hypertension (HTN) (f=8.66, p=.026), patients with clinical anxiety or depression (f=6.20p=.047), and subsequent screw removal (f=6.69, p=.041). PROMIS PI was independently affected by BMI>30 (f=19.29, p=.0046), tobacco use (f=6.26, p=0.0202), coronary artery disease (CAD) (f=12.48, p=.0123), patients with clinical anxiety or depression (f=9.74, p=.0206), and subsequent screw removal (f=20.74, p=.0039). PROMIS depression was independently affected by the presence of CAD (f=6.08, p=.049) and subsequent screw removal (f=9.28, p=.023). Variables influencing PROMIS scores are demonstrated in Table 2. Complications are listed as follows: two of the 15 with subsequent screw removal, two with persistent pain, zero wound dehiscence and three revision surgeries for painful hardware removal were performed.

**Table 2:** PROMIS Scores Based on Demographic and Treatment PROMIS: Patient-reported outcomes measurement information system; PI: Pain interference; PF: Physical function; DM: Diabetes mellitus; HT: Hypothyroidism; HTN: Hypertension; COPD: Chronic obstructive pulmonary disease; CAD: Coronary artery disease; TR/SS: Tightrope/syndesmotic screw

Variable	PROMIS PF	PROMIS PI	PROMIS Depression
	Pr(>F)	Pr(>F)	Pr(>F)
Age	0.643	0.5764	0.211
Sex	0.937	0.6667	0.207
BMI 30+	0.033	0.0046	0.199
ASA 3+	0.766	0.4602	0.117
DM	0.578	0.9973	0.84
Tobacco use	0.00294	0.02024	0.1472
HT	0.653	0.5098	0.8
HTN	0.026	0.1719	0.14
COPD	0.737	0.3333	0.249
CAD	0.051	0.0123	0.049
Anxiety/depression	0.047	0.0206	0.472
Pos. Malleolar fx	0.974	0.6455	0.267
Med. Malleolar fx	0.568	0.2211	0.547
Deltoid reconstruction	0.433	0.5776	0.145
Dislocation	0.196	0.0668	0.166
TR/SS	0.547	0.3262	0.255
Screw rater removed	0.041*	0.0039*	0.023
Revision	0.276	0.59	0.782

Radiographic syndesmotic reduction was assessed at six months postoperatively, on standard weight-bearing radiographs by a foot and ankle fellowship-trained surgeon, as seen in Table 3. Focus was placed on TF clear space and overlap, talocrural angle, medial clear space, Shenton’s line, and fibular station (Table 3). An abnormality in one of these parameters was considered an abnormal reduction for the purposes of this study. A total of 0/24 (0%) patients were malreduced.

**Table 3:** Six Months Post-Operative Syndesmotic Reduction Assessment TF: Tibiofibular

Patient Number	TF Clear Space 1	TF Overlap 1	Medial Clear Space 1	Talocrural Angle 1	Shenton Line 1	TF Clear Space 2	TF Overlap 2	Medial Clear Space 2	Talocrural Angle 2	Shenton Line 2
1	3.8	3.8	3.3	11.1	0	3.3	3	3.3	12.1	0
2	2.7	4.2	2.1	8.5	0	2.6	5.6	2.1	9.5	0
3	2.7	3.6	2.6	11.1	0	2.8	3.9	2.8	14.1	0
4	1.4	5.9	2.1	11.9	0	1.9	5.3	1.9	11.1	0
5	4.6	5.7	4	11.8	0	3.9	3.8	3.3	12.7	0
6	2.5	4.7	2.4	11.3	0	2.9	4.6	2.4	9.7	0
7	2.5	4.7	2.5	11.3	0	2.4	4.5	2.5	9.6	0
8	4	6.8	3.6	8.1	0	3.5	5	3.3	7.5	0
9	3.8	3.2	3.8	8.2	0	4.2	6.2	3.4	6.9	0
10	3.8	3.3	2.4	11.8	0	2.3	3.8	1.8	10.5	0
11	3.2	4.5	2.9	12.3	0	3.4	4.6	3.2	11.4	0
12	3.3	5.3	3.7	12.8	0	2.8	3.8	3.2	12.1	0
13	2.8	5.6	2.8	9.6	0	3.5	4.4	3.6	11.3	0
14	2.2	3.8	2	11.2	0	2.5	3.7	2.2	12.5	0
15	3.2	4.9	3.2	10.8	0	2.9	4.4	2.9	10.1	0
16	3.1	10.8	3.4	11.8	0	4	10	4.2	14.6	0
17	2.9	7.2	2.8	8.5	0	2.5	6.4	2.5	8	0
18	3.6	4.9	3.5	12.8	0	3.8	6.3	3.6	10.7	0
19	3.6	4.6	3.4	12.6	0	4	4.4	3.4	10.4	0
20	2.7	3.7	1.9	12.4	0	3.1	4.4	1.9	10.4	0
21	3.2	4	2.6	11	0	3	3.5	2.5	9.5	0
22	3.4	3.8	3.4	12.6	0	5.3	6.1	3.2	12.5	0
23	3.5	6.3	3	11.8	0	4.6	6.3	3	13.8	0
24	2.7	2.4	3.2	6.9	0	3.2	4	3.4	11.5	0

Table 4 correlates patient characteristics with PROMIS scores. P-values were calculated to demonstrate clinical significance.

**Table 4:** Patient Population Characteristics and Medical History with PROMIS Scores PROMIS: Patient-reported outcomes measurement information system; COPD: Chronic obstructive pulmonary disease; CAD: Coronary artery disease

Variable		PROMIS PI	p-value	PROMIS PF	p-value	PROMIS Depression	p-value
Age	<40	45.48		55.11		43.54	
	>40	44.38	0.73	54.18	0.75	47.8	0.33
Sex	Male	44.8		54.15		48.65	
	Female	44.86	0.99	54.77	0.86	44.71	0.41
BMI	<30	47.41		52.03		43.69	
	>30	41.24	0.07	58.12	0.07	49.29	0.23
ASA Classification	1-2	46.39		54.13		47.31	
	3-4	43.52	0.38	54.94	0.79	44.94	0.58
Diabetes	Yes	42.56		57.54		50.38	
	No	45.44	0.6	53.78	0.52	44.88	0.38
Hypertension	Yes	42.04		56.89		47.91	
	No	46.84	0.16	52.91	0.22	44.68	0.49
COPD	^Yes ^	45.5		50.4		40.15	
	No	44.78	0.86	54.95	0.4	46.56	0.46
CAD	Yes	46.9		52.2		43.6	
	No	44.54	0.79	54.9	0.75	46.37	0.7
Psychiatric condition	Yes	41.3		56.45		47.05	
	No	46.61	0.1	53.62	0.34	45.51	0.76
Posterior malleolus fracture	No	45.69		54.09		45.01	
	Yes	43.14	0.44	55.52	0.65	48.05	0.53
Medial malleolus fracture	No	45.25		54.19		45.59	
	Yes	44.26	0.77	5.09	0.78	46.64	0.81
Deltoid ligament injury	No	45.69		54.15		47.02	
	Yes	41.58	0.27	56.16	0.53	42.24	0.38
Dislocation	No	44.22		55.12		47.12	
	Yes	47.92	0.21	51.83	0.2	40.55	0.29
Repair method	Tight Rope	47.6		53.59		45.37	
	Screw	43.18	0.17	55.15	0.6	46.42	0.81
Screw later removed	No	45.1		54.1		45.21	
	Yes	41.95	0.67	59.75	0.39	55	0
Pain 2+ years	No	45.1		54.1		45.21	
	Yes	41.95	0.67	59.75	0.39	55	0
Revision	No	44.81		54.37		45.51	
	Yes	45.03	0.97	55.97	0.76	49.6	0.54

## Discussion

MFs are important to properly diagnose and treat for achieving optimal outcomes. Operative intervention, through various fixation techniques, has historically produced the best outcomes for MF [6]. However, no studies have evaluated outcomes utilizing PROMIS scores. PROMIS scores are more advantageous than other data collection methods when gathering post operative data. Orthopedic studies, including those of MFs, benefit from PROMIS score calculations because PROMIS scores utilize the patient's voice and perspective on the operation's success by assessing their pain, improvement, psychiatric condition, PF, and other variables. Our study, the first utilizing PROMIS, found mean postoperative PROMIS scores for PF, PI, and depression to be 44.83, 54.56, and 46.03, respectively. Patient-reported outcome measures were collected at a minimum of two years and radiographic outcomes were assessed only at the six-month follow-up visit for this study. The reported outcomes indicate that patients can expect to be within +/- 1 standard deviation of the population mean for PF, pain, and depression following operative fixation of this injury. Furthermore, all patients were adequately reduced on six-month weight-bearing radiographs, therefore, limiting any analysis on the impact malreduction had on patient-reported outcome measures. Interestingly, the presence of concomitant injuries to the medial malleolus, posterior malleolus, and/or deltoid ligament did not significantly impact PROMIS scores.

Our study found that the method of surgical intervention, fixation with SS (15 patients, 62.5%) or suture button (nine patients, 37.5%), did not significantly impact PROMIS scores. The literature largely supports our findings. Raedar et al., in a randomized control trial, found no significant differences in various patient-reported outcome measures (American Orthopaedic Foot and Ankle Society (AOFAS) hindfoot, Manchester, Oxford Foot Questionnaire, and Molander Ankle Score) following syndesmotic fixation between suture buttons and SSs [7]. Raedar et al.'s data was limited to patients from one rural hospital outpatient service. Zhang et al., in a systematic review in 2017, compared suture button and SS fixation and concluded the functional outcomes (AOFAS) between the two types of fixations were not significantly different. However, SSs did have higher rates of implant failure, hardware removal surgeries, and lower rates of anatomic reduction [8]. Lehtola et al., in an analysis of Weber C fractures, found comparable functional outcomes and maintenance of reduction at a six-year follow-up [9]. Although these patients were not MFs, this study supports the efficacy of both methods of fixation following a similar syndesmotic injury.

The presence of a posterior malleolus, medial malleolus, deltoid ligament injury requiring surgical intervention, or those with a dislocation at initial presentation did not significantly impact patient-reported outcomes. The authors initially hypothesized concomitant ankle injuries would have a significant negative impact on PROMIS scores. Although previous studies have demonstrated no difference in functional status in the short-term following Weber B vs Weber C fracture patterns, the association between ankle fracture severity and functional outcomes continues to be a topic of debate [1,10-12]. Our study indicates that the presence of concomitant injuries does not significantly affect patient-reported outcomes in this specific population subset, however, more studies are needed to determine the true correlation.

Radiological assessment of accurate anatomic reduction of syndesmosis is of paramount importance. Fibular shortening can result in lateral talar shift and increase abnormal joint reactive forces; Ramsey et al. and Lloyd et al. found that a 1 mm shift can reduce weight-bearing portion by 42%, leading to early post-traumatic osteoarthritis [13-15]. Traditionally, syndesmosis reduction is evaluated in static and stressed anterior-posterior (AP) and mortise views. A cadaver study conducted by Harper et al. concluded that TF “clear space” less than 6 mm on the AP and mortise view, TF overlap greater than 6 mm or 42% of the fibular width on the AP view, and TF overlap on the mortise view greater than 1 mm are considered to be normal values [16]. The width of the TF “clear space” on both AP and mortise views was determined to be the most reliable parameter for detecting early syndesmotic widening [16]. However, studies have disputed the utility of the various radiographic parameters of the distal TF syndesmosis, as most of the values are highly dependent on the proper positioning of the ankle. Evaluation of fibular station in lateral radiographs is often underutilized when assessing the syndesmotic reduction. Grenier et al. described the anteroposterior TF (APTF) ratio, which is normally 0.94 ± 0.13, as a tool to identify malreduction of the fibula in the incisura fibularis [13]. Another cadaver study noted that standard radiographs can only identify syndesmotic reduction with an accuracy of 4 mm, whereas a CT scan can identify syndesmotic widening at 2 mm diastasis [17]. Recent studies noted that more than 50% of syndesmosis were malreduced on CT evaluation. However, using intraoperative CT scans for assessing syndesmotic reduction is not feasible for multiple facilities because of the financial restraints. Summers et al. accurately evaluated syndesmotic reduction based on contralateral radiographs and advised that using standard numbers for assessing syndesmotic reduction is not accurate owing to anatomical variations and individual patient size discrepancies [18].

Obesity and its negative impact on outcomes in foot and ankle orthopedics have been heavily mentioned in the literature [19]. Mendelsohn et al., in a study of over 200 patients, demonstrated obese patients had a 12-fold increased risk for loss of reduction following syndesmotic ankle fixation compared to nonobese patients [19]. Excess BMI in patients undergoing open reduction internal fixation (ORIF) for ankle fractures has been shown to negatively impact both PROMIS PF and PROMIS PI [20]. These findings are likely a result of excessive loading on the fixation site and an increase in baseline inflammatory markers such as interleukin-6, CRP, and leptin [20]. These prior studies validate our findings that patients with BMI>30 have a lower PF (p=0.033) and higher pain (p=0.0046) than those with BMI<30 (Table 4).

Tobacco use has been cited as a factor negatively impacting outcomes following foot and ankle procedures [21-24]. Audet et al., in a study of 900 patients, recently described tobacco dependence to be an independent predictor for worse foot functional index (FFI) scores and prescription pain medication usage following ankle fracture procedures [21]. Heyes et al., in a 2021 systematic review, concluded that tobacco use negatively impacts pain control and functional outcomes following foot and ankle procedures [23]. Similarly, a meta-analysis by Pearson et al. found smokers took 27.6 more days to unite a fracture and were 2.2 times more likely to have a nonunion [24]. In our study, smoking was independently associated with significantly higher PROMIS PI (p=0.2020) and lower PROMIS PF (p=0.0029) (Table 4). Negative outcomes, and their relationship with tobacco use, are likely due to the systemic inflammation, neuropathy, and vascular damage caused by smoking [25].

Diabetes mellitus (DM) and its impact on patient outcomes following foot and ankle surgery is another well-documented topic in foot and ankle literature [26]. Egol et al. found an absence of type 2 DM (DM2) to significantly increase the chance of recovering PF one year following surgical management of an ankle fracture [10]. However, McKissack et al. found no significant associations between postoperative complications following ORIF of ankle fractures in patients with DM2 [11]. Our linear regression analysis did not find the diagnosis of DM2 to independently influence PROMIS scores in MF. One hypothesis for the discrepancies found in the literature is that patients with DM2 are at higher risk for more chronic medical conditions and comorbidities than their counterparts, which can lead to confounding results [27]. In our study, while controlling for these other comorbidities, we concluded that DM2 did not significantly impact the outcomes, although future prospective studies that independently isolate DM2 are needed to assess the true impact.

Psychiatric conditions, such as anxiety and depression, have been shown to confer worse outcomes following orthopedic procedures. Russo et al. demonstrated patients with depression had lower function when performing activities of daily living and strength testing [28]. Mehta et al. drew similar conclusions and found that patients with clinical anxiety reported lower levels of self-reported functioning [14]. This combination of findings in the literature suggests that patients with psychiatric conditions have lower baseline levels of perceived and actual PF. These prior findings are supported by our data, which shows that psychiatric conditions have a significant negative impact on both PF and pain. Preoperative PROMIS depression scores would be beneficial in evaluating the extent of individual patient limitations preoperatively and would allow us to better assess any potential improvement resulting from surgery.

Hypertension and its impact on surgical outcomes have been thoroughly studied. Costigan et al.'s study on patients undergoing ORIF for ankle fractures and Baker et al.’s study on total knee arthroplasty both found significant increases in complication rates among hypertensive patients [29]. Although different patient outcomes were measured, the trend of HTN negatively affecting overall postoperative success is supported by our results, which found HTN to independently impact PROMIS PF. Similarly, the presence of CAD independently influenced higher PROMIS PI scores. Our results, shown in Table 2, suggest cardiovascular risk factors should be taken into consideration when treating MF, especially when counseling patients on expected outcomes regarding PF and pain management.

Our study had two patients who underwent SS removal for persistent pain over the lateral malleolus and both patients scored significantly worse in all PROMIS domains. Hardware is a known cause of persistent pain following ankle fracture surgery and hardware removal has been shown to statistically improve patients’ pain and PF when compared to their own baseline [30]. It is difficult to draw conclusions based on two hardware removals and further studies are needed to analyze the true impact on patient-reported outcomes.

This study is not without limitations. Its retrospective design, lack of randomization, and small sample size due to the rarity of MF presentation were all limitations. The small sample size limits the ability to draw strong conclusions between certain patient comorbidities and injury characteristics with reported outcomes. Additionally, selection bias may have been present based on physician preferences for certain fixation techniques. These preferences were not something that could be tangibly measured. Our study also lacked scheduled follow-up radiographs at the time PROMIS scores were collected, limiting our ability to assess the permanence of the reduction and any potential impact on outcome measures. Long-term radiographs should be included in further studies as outcome measures. Multicenter studies would be ideal in order to increase the cohort size and allow better analysis of the impact radiographic findings and patient characteristics have on self-reported outcomes following MF.

## Conclusions

In conclusion, patients with surgically managed MFs can expect to return within one standard deviation of the population mean for PROMIS PF, pain, and depression. This is a novel study that serves an important purpose for patients undergoing operative fixation for MF. By highlighting radiographic and patient characteristics that influence patient-reported outcomes, physicians will have the ability to appropriately manage patient expectations prior to surgery. This study can serve as one of the first prognostic tools for physicians operating on Maisonneuve injuries.
